# ADAM Metallopeptidase domain 19 promotes skin fibrosis in systemic sclerosis via neuregulin-1

**DOI:** 10.1186/s10020-024-01047-8

**Published:** 2024-12-23

**Authors:** Qiming Meng, Ding Bao, Sijia Liu, Jing Huang, Muyao Guo, Bingying Dai, Liqing Ding, Shasha Xie, Meng Meng, Chunliu Lv, Weijia He, Hui Luo, Honglin Zhu

**Affiliations:** 1 Department of Rheumatology and Immunology, Xiangya Hospital, Central South University, Changsha, Hunan 410008 P.R. China; 2 Provincial Clinical Research Center for Rheumatic and Immunologic Diseases, Xiangya Hospital, Changsha, Hunan 410008 P.R. China; 3 National Clinical Research Center for Geriatric Disorders, Xiangya Hospital, Central South University, Changsha, Hunan 410008 P.R. China; 4 Department of Pathology, Xiangya Hospital, Changsha, 410008 P.R. China; 5 Department of Breast Tumor Plastic Surgery, The Affiliated Cancer Hospital of Xiangya School of Medicine, Hunan Cancer Hospital, Central South University, 283 Tongzipo Road, Changsha, Hunan 410013 P.R. China

**Keywords:** ADAM19, Dermal fibroblasts, Systemic sclerosis, TGF-β, NRG1

## Abstract

**Background:**

ADAM19 (ADAM Metallopeptidase Domain 19) is known to be involved in extracellular matrix (ECM) remodeling, yet its specific function in systemic sclerosis (SSc) fibrosis remains unclear.

**Objectives:**

This study sought to clarify the role and underlying mechanism of ADAM19 in SSc skin fibrosis.

**Methods:**

The expression of ADAM19 was assessed in skin tissues of SSc and wound healing using publicly available transcriptome datasets. This analysis was further validated through real-time PCR, western blot, and immunostaining in our SSc cohort, as well as in a mouse model of hypochlorite (HOCl)-induced fibrosis. To downregulate the expression of ADAM19, ADAM19 siRNA was employed. The influence of ADAM19 on fibroblast transcriptomics was examined using bulk RNA-seq. Data analysis and visualization were conducted using R packages, including edgeR, limma, clusterProfiler, ggplot2, gseaplot2, and complexheatmap.

**Results:**

ADAM19 exhibited a significant upregulation in skin tissues of SSc patients, as well as in wound healing and a HOCl-induced fibrosis mouse model. Additionally, there was a notable positive correlation between ADAM19 and fibrosis-related genes, local skin score, Modified Rodnan skin score, skin thickness progression rate, and the presence of ARA antibodies in SSc patients. Furthermore, ADAM19 levels were markedly elevated in SSc primary dermal fibroblasts and TGF-β-stimulated healthy controls primary dermal fibroblasts. The downregulation of ADAM19 resulted in the repression of TGF-β-induced ECM deposition and fibroblast activation. ADAM19 was identified as a mediator for the shedding of neuregulin-1 (NRG1) in fibroblasts, a pro-fibrotic cytokine that must be cleaved to exert its function.

**Conclusion:**

ADAM19 plays a role in TGF-β-induced ECM deposition and fibroblast activation by mediating the shedding of NRG1, ultimately contributing to the development of skin fibrosis in SSc.

**Supplementary Information:**

The online version contains supplementary material available at 10.1186/s10020-024-01047-8.

## Introduction

Systemic sclerosis (SSc) is an immune-mediated connective tissue disease characterized by persistent activation of the immune system, vascular injury, and excessive deposition of extracellular matrix (ECM), resulting in fibrosis (Prescott et al. 1992; Fleischmajer et al. 1977; Kähäri et al. 1988). The main clinical presentations of SSc include skin and lung fibrosis, renal crisis, Raynaud’s phenomenon, pulmonary arterial hypertension, and gastrointestinal complications (Volkmann et al. 2022). The molecular mechanisms underlying skin fibrosis in SSc remain unclear but are likely to involve several molecules and signaling pathways, with the TGF-β signaling pathway playing a central role (Varga and Pasche 2009; Heim et al. 2022; Wei et al. 2012; Denton et al. 2018; Dees et al. 2011). TGF-β can activate fibroblast and promote myofibroblast differentiation, leading to increased ECM deposition. The deposition of ECM and the fibroblast to myofibroblast transdifferentiation were closely associated with the severity of clinical disease in SSc skin (Kissin et al. 2006).

A disintegrin and metalloproteinase (ADAMs) are transmembrane proteins with multiple domains that serve crucial roles in cell fate determination, cell migration, cell proliferation, heart development, immunity, and wound healing (Reiss and Saftig 2009; Edwards et al. 2008). ADAM17 and ADAM12 are two of the most extensively studied members of the ADAM family of proteins. ADAM17 plays crucial role in the shedding activity of cell surface molecules, which involves cleavage of TNF-α, IL-6R, EGFR ligands, and Notch receptors. It also coordinates immune and inflammatory responses (Zunke and Rose-John 2017; Murthy et al. 2012). The main focus of developing pharmaceutical inhibitors for ADAM17 was its effect on TNF-α in rheumatoid arthritis (DasGupta et al. 2009). ADAM12 is vital in the inflammatory-induced synovial membrane, skin, and lung fibrosis. TGF-β induces the expression of ADAM12 and promotes TGF-β depending signaling through interaction with the type II receptor of TGF-β (Cipriani et al. 2016; Atfi et al. 2007; Kerna et al. 2014).

ADAM19 contains all functional domains of ADAMs. These include a disintegrin, a cysteine-rich region, a signal peptide, a propeptide, an epidermal growth factor (EGF)-like domain, a transmembrane sequence, a metalloprotease, and a cytoplasmic tail domain (Qi et al. 2009). ADAM19 cleaves EGF-like growth factors, such as heparin-binding (HB)-EGF and neuregulin (NRG), from the cell membrane through shedding activity (Horiuchi et al. 2005; Shirakabe et al. 2001). Mice deficient in ADAM19 exhibit cardiac dysplasia, with the majority succumbing shortly after birth. This underscores the pivotal role of ADAM19 in development (Zhou et al. 2004). Furthermore, ADAM19 is often overexpressed in various fibrotic conditions, such as pulmonary, renal, and cardiac fibrosis (Keating et al. 2006; Ramdas et al. 2013; Gao et al. 2020). However, the exact role of ADAM19 in fibrotic diseases is still unknown and requires further research.

In this study, we investigated the expression levels of ADAM19 in the skin tissues of patients with SSc and a mouse model of hypochlorite (HOCl)-induced fibrosis. Furthermore, we aimed to elucidate the functional role of ADAM19 in skin fibrosis associated with SSc.

## Materials and methods

### Gene expression omnibus (GEO) datasets analysis

The R packages limma and edgeR were employed to identify significantly differentially expressed genes (DEGs) in four datasets: GSE130955 (58 SSc patients and 33 healthy controls (HC), GSE58095 (59 SSc patients and 43 HC), GSE181549 (112 SSc patients and 44 HC), and GSE124161 (wound healing samples at day 0 and weeks 1, 2, 3, 4, 5, 6, and 8). Single-cell RNA-seq (scRNA-seq) data Bam files and the raw matrix of skin tissue samples from 12 SSc patients and 10 healthy controls were retrieved from the GEO database (GSE138669). Data analysis was conducted using Seurat package V4.0.0, wherein cells expressing fewer than 300 genes, 600 unique molecular identifiers (UMIs), or more than 20% mitochondrial genes were filtered out. Sample integration was performed using SCTransform and canonical correlation analysis (CCA). Principal component analysis (PCA) and uniform manifold approximation and projection (UMAP) were utilized for data visualization. Manual annotation of main clusters was done using canonical markers. Based on previous research, fibroblast clusters, identified by COL1A1, COL1A2, and PDGFRA, were reclustered into 10 distinct clusters: ANGPTL7, CCL19/C7/APOE_hi, CRABP1 (DP), COL11A1 (DS), POSTN/ASPN, MYOC/FMO1/APOE_low, PCOLCE2, SFRP2/PRSS23, SFRP2/SFRP4, and SFRP2/WIF1 (Tabib et al. 2021). Visualization was performed using R packages clusterprofiles, ggplot2, and complexheatmap.

### Patients and samples

Six newly diagnosed and untreated SSc patients were enrolled in the Department of Rheumatology and Immunology at Xiangya Hospital, Central South University, in Changsha, Hunan, China. All patients met the 2013 ACR/EULAR criteria for SSc. Skin samples were taken from the outer forearm, 8 cm from the proximal tip of the styloid process of the ulna. Additionally, six HC, matched for age and sex, were included to procure skin tissues. Before enrollment, written informed consent was obtained from all patients and healthy controls. The clinical characteristics of the SSc patients are detailed in Table S1.

### HOCl-induced fibrosis mouse model

Six-week-old female BALB/c mice were obtained from SJA Laboratory (Hunan, China) and were randomly assigned to experimental (*n* = 7) and control groups (*n* = 6). HOCl was generated by adding NaClO solution (active chlorine concentration of 6–10%) to KH2PO4 solution (100nM; pH 6.2) at dilutions (1:110 NaCLO: KH2PO4). A total of 100uL of the diluted HOCl solution was prepared temporarily and subcutaneously injected every day for a duration of 6 weeks into the pre-shaved back of mice. The control group of mice received injections of 100uL of sterilized PBS. Skin samples were taken from the injected back skin.

### Immunohistochemical analysis

The expression levels of ADAM19 were evaluated through immunohistochemistry in the skin samples from groups including HC, SSc patients, phosphate-buffered saline (PBS) controls, and a hypochlorite (HOCl)-induced fibrosis mouse model. Formalin-fixed paraffin-embedded (FFPE) tissue sections were deparaffinized and subjected to antigen retrieval using a citric acid repair buffer. After inhibiting endogenous peroxidase activity with 3% H2O2 for 25 min, nonspecific binding was blocked for 30 min using 3% bovine serum albumin (BSA). Slides were then incubated overnight at 4℃ with antibodies against ADAM19 (ab191457, Abcam, China) at a dilution of 1:500. Following this, the slides were treated with an enzyme-labeled goat anti-rabbit antibody (Servicebio, China) at room temperature for 50 min. Subsequently, they were visualized using a diaminobenzidine kit as a chromogenic agent. All slides were observed using an Olympus microscope, and images were captured at 200× magnification with a digital camera.

### Cell culture, stimulation, and transfection

Human primary dermal fibroblasts were isolated from the skin of six SSc patients and six age- and sex-matched HC. Skin samples were taken from the outer forearm, 8 cm from the proximal tip of the styloid process of the ulna. These fibroblasts were cultured in DMEM F-12 supplemented with 10% FBS and 1% penicillin/streptomycin at 37℃ with 5% CO2. For all experiments, fibroblasts within passages 4–8 were utilized. The human primary dermal fibroblasts isolated from HC were stimulated with recombinant human TGF-β (10 ng/ml) (PeproTech, Cranbury, NY, USA) 48 h. Cells were re-stimulated with TGF-β every 24 h to maintain high levels of TGF-β.

HC primary dermal fibroblasts were then transfected with 100 nM si-h-ADAM19_002 (si-ADAM19) (RiboBio, Guangzhou, China) using the Lipofectamine 3000 kit (Invitrogen, USA) following the manufacturer’s protocol. The control group was the fibroblasts transfected with negative control siRNA (si-NC) (RiboBio, Guangzhou, China). Si-ADAM19 or si-NC was supplemented into the 0.5% FBS cell culture medium without penicillin/streptomycin and then incubated with dermal fibroblasts. After 24 h, the cell culture medium was replaced with 0.5% FBS culture medium containing TGF-β to stimulate fibroblasts for 48 h. Cells were subsequently re-stimulated with TGF-β every 24 h to maintain elevated TGF-β levels.

### Fibroblast treatment with recombinant human NRG1

HC primary dermal fibroblasts were subjected to a 24 h serum starvation, followed by adding 50 ng/ml recombinant human NRG1 (396-HB-050, R&D Systems, USA) for an additional 24 h.

### Quantitative real time-PCR

Total RNA was extracted utilizing the TRIzol reagent following the manufacturer’s instructions. The concentration and quality of RNA were assessed using a QS3000 spectrophotometer. For quantitative real-time PCR (qRT-PCR), gene-specific primers were employed with SYBR Green (SYBR Premix Ex Taq RT-PCR kit, Takara) on a 7500 Real-Time PCR System (Applied Biosystems, Waltham, MA, United States). The primer sequences can be found in Table S2. GAPDH was employed as the internal reference, and the 2^−ΔΔCt^ method was used to quantify the relative expression of mRNA. Each qRT-PCR reaction was performed with at least three biological and technical replicates.

### Western blot analysis

Total protein was extracted from the cultured human primary dermal fibroblasts using RIPA lysate buffer supplemented with a protease/phosphatase inhibitor cocktail (Beyotime, Shanghai, China). BCA protein detection kits (Cat#. BCA02, DingGuo, China) were used to determine the protein concentrations of each group. The same amounts of protein were separated using 10% SDS-PAGE and transferred to polyvinylidene fluoride (PVDF) membranes. Membranes were blocked with 5% skim milk powder in 0.1% Tween’s Tris-buffer saline for 2 h, and subsequently incubated with specific primary antibodies against ADAM19 (ab191457, Abcam, China), ACTA2 (ab5694, Abcam, China), NRG1 (A0687, ABclonal, China), and GAPDH (#2118, CST, China) overnight at 4 ℃. Then the anti-rabbit IgG enzyme-conjugated secondary antibody (Proteintech, Wuhan, China) was incubated as room temperature for 1 h. Enhanced chemiluminescence (Bio-RAD, Hercules, CA, USA) was used to detect western blot results.

### The measurement of soluble NRG1 levels in dermal fibroblast supernatant

Soluble NRG1 (sNRG1) levels in cultured human primary dermal fibroblasts supernatants were assessed using ELISA (DY377, R&D Systems, USA) by the manufacturer’s protocol.

### Library construction for RNA sequencing and bioinformatic analysis

Total RNA was extracted from cultured human primary dermal fibroblasts obtained from HC, both with or without ADAM19 knockdown, and stimulated with or without recombinant TGFβ. RNA-seq was performed by Novogene (Beijing, China) on an Illumina NovaSeq platform with a paired-end 150 bp sequencing strategy. The raw sequencing data were trimmed to remove adapters, low-quality bases, and other contaminants. Alignment and mapping of sequencing reads were performed using Hisat2 (v2.1.0). The count data was normalized using the “TMM” methodology. The Bioconductor package edgeR was used for differential expression analyses. Differentially expressed genes (DEGs) were considered significant when p-values < 0.05. The results are visualized through Volcano blots and heatmaps using the R package ggplot2. Gene set enrichment analyses (GSEA) were performed using the R package clusterProfiler and visualized by gseaplot2.

### Statistical analysis

Data analysis was performed using GraphPad Prism version 9.0. Paired or unpaired t-tests were performed for parametric data. For nonparametric data, the Mann-Whitney U test was used. ANOVA test was used in multiple comparisons. Quantitative data are expressed as mean ± SEM. Spearman’s correlation analysis was utilized to examine correlations. P-values less than 0.05 were considered statistically significant.

## Results

### ADAM19 expression in skin tissues of SSc cohorts and its correlation with clinical indices

**Fig. 1:**
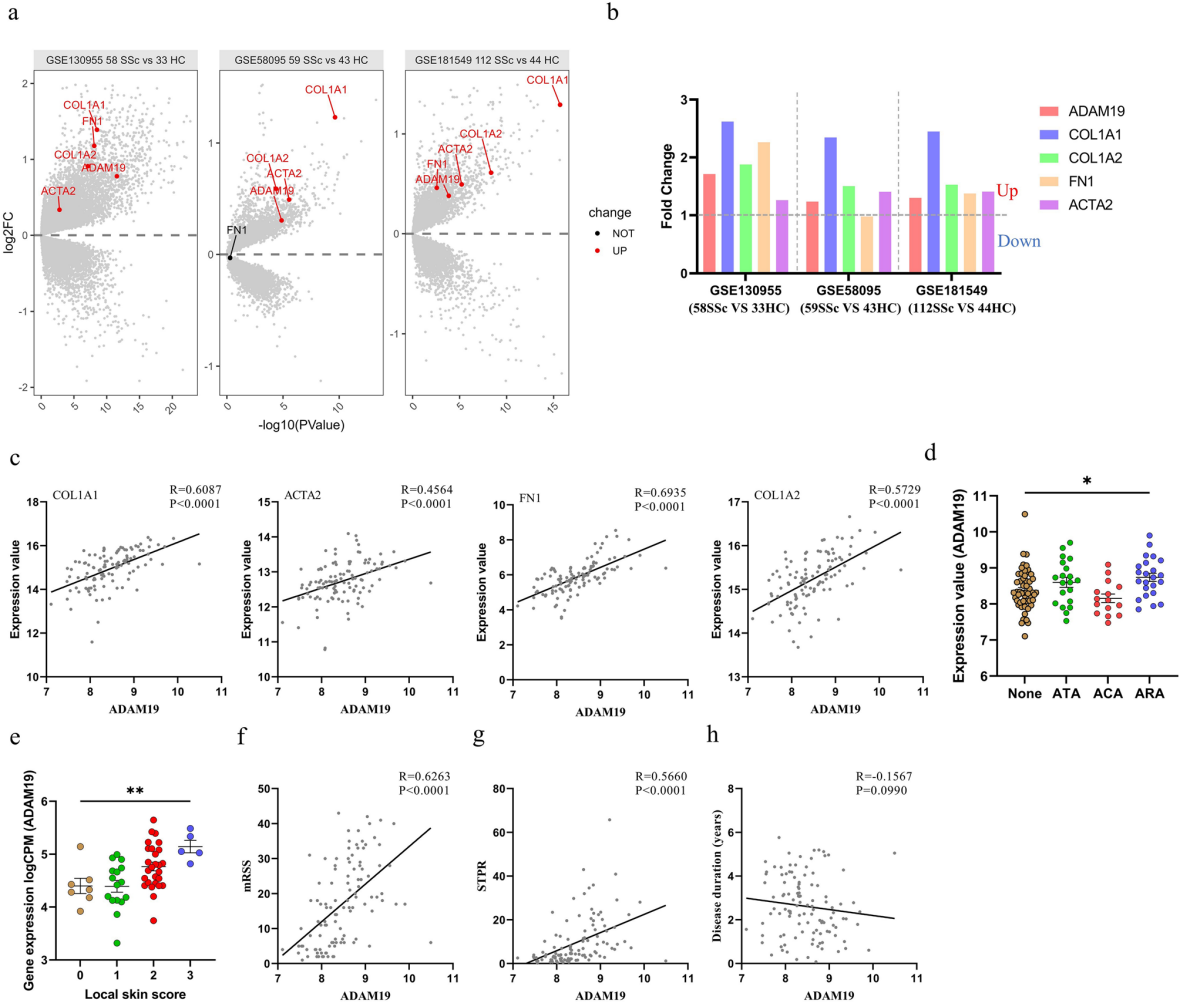
ADAM19 expression in skin tissues of SSc patients from GEO datasets. (**a**) Volcano plots displaying ADAM19 and fibrosis-related genes (ACTA2, COL1A1, COL1A2, and FN1) expression in SSc skin GEO datasets (GSE130955, GSE58095, GSE181549). (**b**) Fold change ratio for ADAM19 and fibrosis-related genes (ACTA2, COL1A1, COL1A2, and FN1) in SSc skin GEO datasets (GSE130955, GSE58095, GSE181549). (**c**) Correlation between ADAM19 expression and fibrosis-related genes (ACTA2, COL1A1, COL1A2, and FN1) expression in SSc patients (GSE181549). (**d**) ADAM19 gene expression levels in skin tissues of SSc patients with negative antibody, and positive for ATA, ACA, and ARA (GSE181549). (**e**) ADAM19 gene expression levels in skin tissues of SSc patients with different local skin scores (GSE130955). (**f**) Correlation between ADAM19 expression and mRSS in SSc patients (GSE181549). (**g**) Correlation between ADAM19 expression and STPR in SSc patients (GSE181549). (**h**) Correlation between ADAM19 expression and disease duration (years) in SSc patients (GSE181549). SSc, systemic sclerosis; HC, healthy controls; mRSS, modified Rodnan Skin Score; STPR, skin thickness progression rate; ATA, antibodies to DNA topoisomerase I; ARA, antibodies to RNA polymerases; ACA, antibodies to centromere; red color, up-regulated genes; blue color, down-regulated genes. Data are represented as mean ± SEM. **, *P* < 0.01. ****, *P* < 0.0001

We initially assessed the expression levels of ADAM19 in the transcriptome data of three SSc cohorts, namely the PRSS cohort (GSE130955, comprising 58 SSc patients and 33 healthy controls), and GENISOS cohort (GSE58095, including 59 SSc patients and 43 healthy controls; GSE181549, consisting of 112 SSc patients and 44 healthy controls). We observed an upregulation of ADAM19 and fibrosis-related genes (COL1A1, COL1A2, and ACTA2) in the skin tissues of SSc patients compared to HC (Fig. 1a, b). Following this, we explored the relationships between ADAM19 expression and fibrosis-related indices, and clinical features of SSc. Correlation analysis unveiled a noteworthy positive correlation between ADAM19 expression levels and fibrosis-related genes (Fig. 1c). SSc patients with positive anti-RNA polymerase III antibodies (ARA) demonstrated significantly higher ADAM19 expression levels than those with negative autoantibodies (Fig. 1d). ADAM19 exhibited positive correlations with local skin scores, modified Rodnan skin scores (mRSS), and skin thickness progression rate (STPR). However, there was no correlation between ADAM19 and disease duration (Fig. 1e-h). Moreover, we observed that the expression levels of ADAM19 in the skin were considerably higher in SSc patients with ILD, as compared to those without ILD (Figure S1a). Skin ADAM19 levels were negatively correlated with forced vital capacity (FVC; % predicted) and diffusing capacity for carbon monoxide (DLco; % predicted) (Figure S1b, c).

Additionally, we delved into the expression levels of ADAM19 in wound healing, a classical skin fibrotic disease. Upon analyzing transcriptome data (GSE124161) at different stages of wound healing, we observed a significant upregulation of ADAM19 in weeks 1, 2, 3, 4, 5, 6, and 8 compared to day 0 (Figure S2a). The expression of ADAM19 paralleled the expression of fibrosis-related genes (COL1A1, COL1A2, ACTA2, and FN1) (Figure S2b).

### Validating ADAM19 expression in SSc skin tissues and experimental fibrosis models

**Fig. 2:**
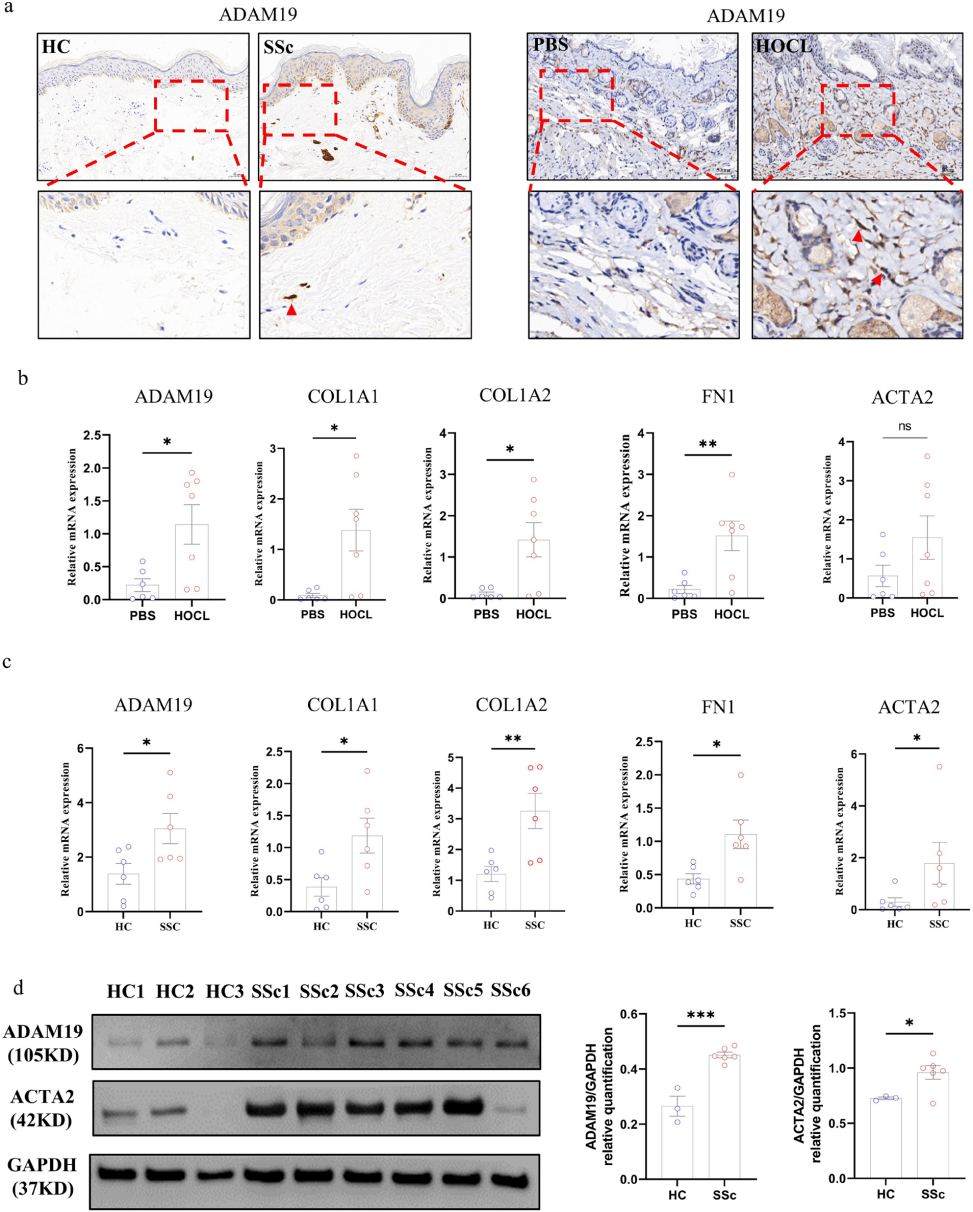
ADAM19 expression in skin tissues of SSc patients and HOCl-induced experimental models of fibrosis. (**a**) Expression levels of the ADAM19 protein in skin tissues of HC, SSc patients, PBS, and HOCl induced fibrosis mouse model. (**b**) Expression levels of the ADAM19 and fibrosis-related genes (ACTA2, COL1A1, COL1A2, and FN1) mRNA in skin tissues of PBS and HOCl-induced fibrosis mouse model. (**c**) Expression levels of the ADAM19 and fibrosis-related genes (ACTA2, COL1A1, COL1A2, and FN1) mRNA in isolated primary dermal fibroblasts of HC and SSc patients. (**d**) Expression levels of the ADAM19 and ACTA2 protein in isolated primary dermal fibroblasts of HC and SSc patients. SSc, systemic sclerosis; HC, healthy controls; HOCl, hypochlorous acid; PBS, phosphate buffer saline. Data are represented as mean ± SEM. *, *P* < 0.05, **, *P* < 0.01. ****, *P* < 0.0001

Further analysis of ADAM19 expression in human and mouse fibrotic skin tissues was conducted. Immunohistochemistry revealed an elevated expression of ADAM19 in the fibroblasts of skin tissues from SSc patients and the HOCl-induced fibrosis mouse model compared to the control group (Fig. 2a). RT-PCR also demonstrated a substantial upregulation in the mRNA expression levels of ADAM19 and fibrosis-related genes (COL1A1, COL1A2, and FN1) in the skin tissues of the HOCl-induced fibrosis mouse model compared to controls (Fig. 2b). Additionally, we explored whether the expression of ADAM19 was specific to certain fibroblast subtypes. We further analyzed the expression of ADAM19 in fibroblast subtypes from the single-cell RNA sequencing transcriptome data of SSc skin tissues (GSE138669). A noteworthy increase in ADAM19 expression was observed in COL11A1^+^ fibroblasts in SSc compared to HC. COL11A1^+^ fibroblasts were shown to be a profibrotic fibroblast subtypes in published data (Figure S3a) (Tabib et al. 2021; Xue et al. 2022). In primary dermal fibroblasts, SSc exhibited increased mRNA expression levels of ADAM19 and fibrosis-related genes (ACTA2, COL1A1, COL1A2, and FN1). The protein expression of ADAM19 and ACTA2 was also significantly elevated (Fig. 2c-d).

### The role of ADAM19 in TGF-β-induced fibrosis

**Fig. 3:**
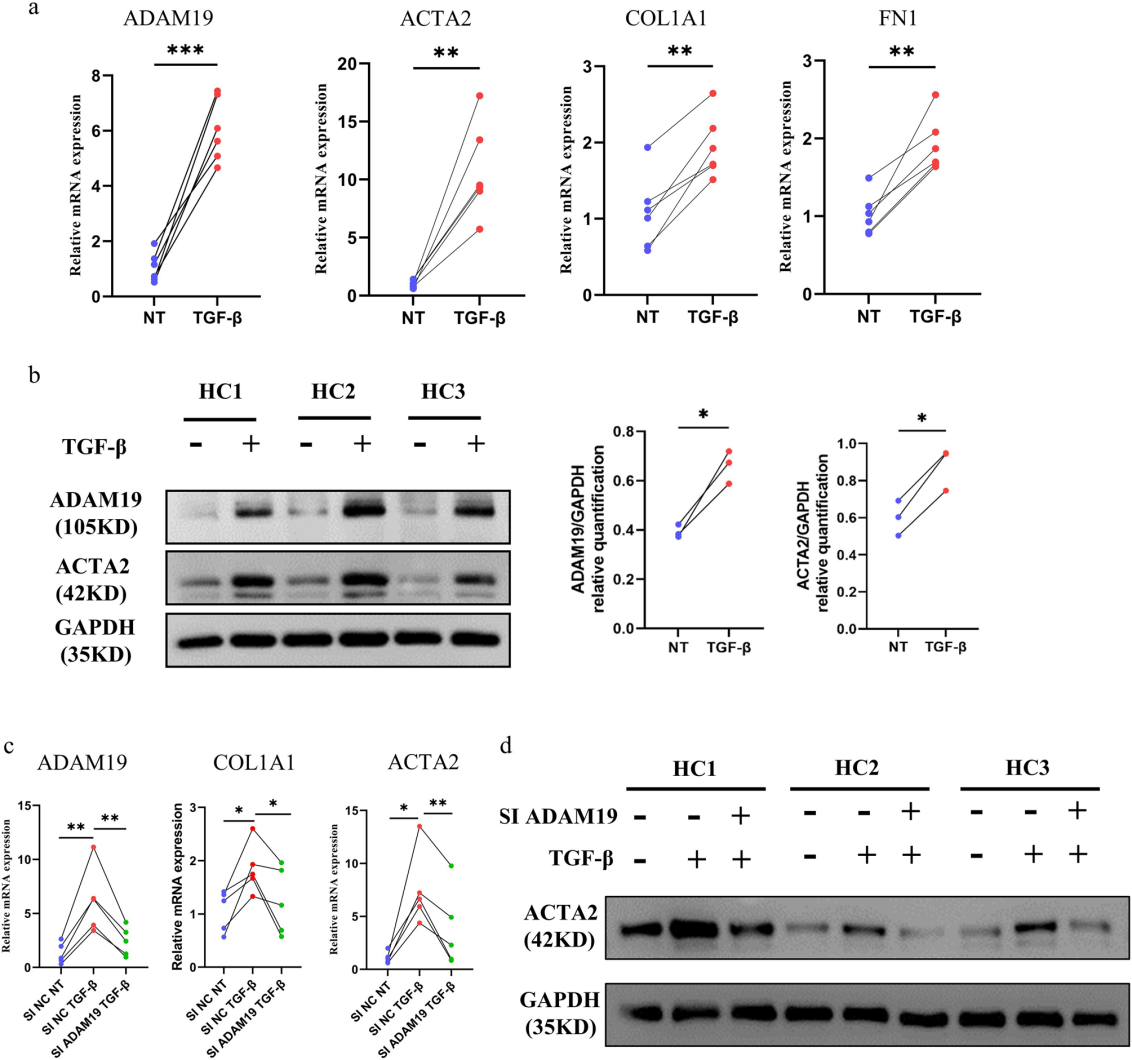
Suppression of ADAM19 expression inhibited the TGF-β induced fibrosis. (**a**) mRNA expression of ADAM19 and fibrosis-related genes, including ACTA2, COL1A1, and FN1 in human primary dermal fibroblasts isolated from HC incubated with TGF-β (10ng/ml) 48 h. (**b**) Protein levels of ADAM19 and ACTA2 in human primary dermal fibroblasts isolated from HC incubated with TGF-β (10ng/ml) 48 h. (**c**) mRNA expression of ADAM19, COL1A1, and ACTA2 in human primary dermal fibroblasts isolated from HC transfected with negative control siRNA and ADAM19 siRNA with or without TGF-β (10ng/ml) stimulation for 48 h. (**d**) Protein expression of ACTA2 in human primary dermal fibroblasts isolated from HC transfected with negative control siRNA and ADAM19 siRNA with or without TGF-β (10ng/ml) stimulation for 48 h. HC, healthy controls. *, *P* < 0.05, **, *P* < 0.01. ***, *P* < 0.001

After incubation with recombinant human TGF-β for 48 h, the mRNA levels of ADAM19 and fibrosis-related genes (COL1A1, ACTA2, and FN1) increased in HC primary dermal fibroblasts cultured in vitro (Fig. 3a). The protein levels of ADAM19 and ACTA2 were also significantly upregulated (Fig. 3b). Further, we examined whether the knockdown of ADAM19 can inhibit TGF-β-induced fibrosis and fibroblast activation. Fibroblasts with knocked-down ADAM19 displayed reduced sensitivity to TGF-β-induced profibrotic effects, as demonstrated by a notable decrease the levels of the COL1A1 and ACTA2 (Fig. 3c, d).

**Fig. 4:**
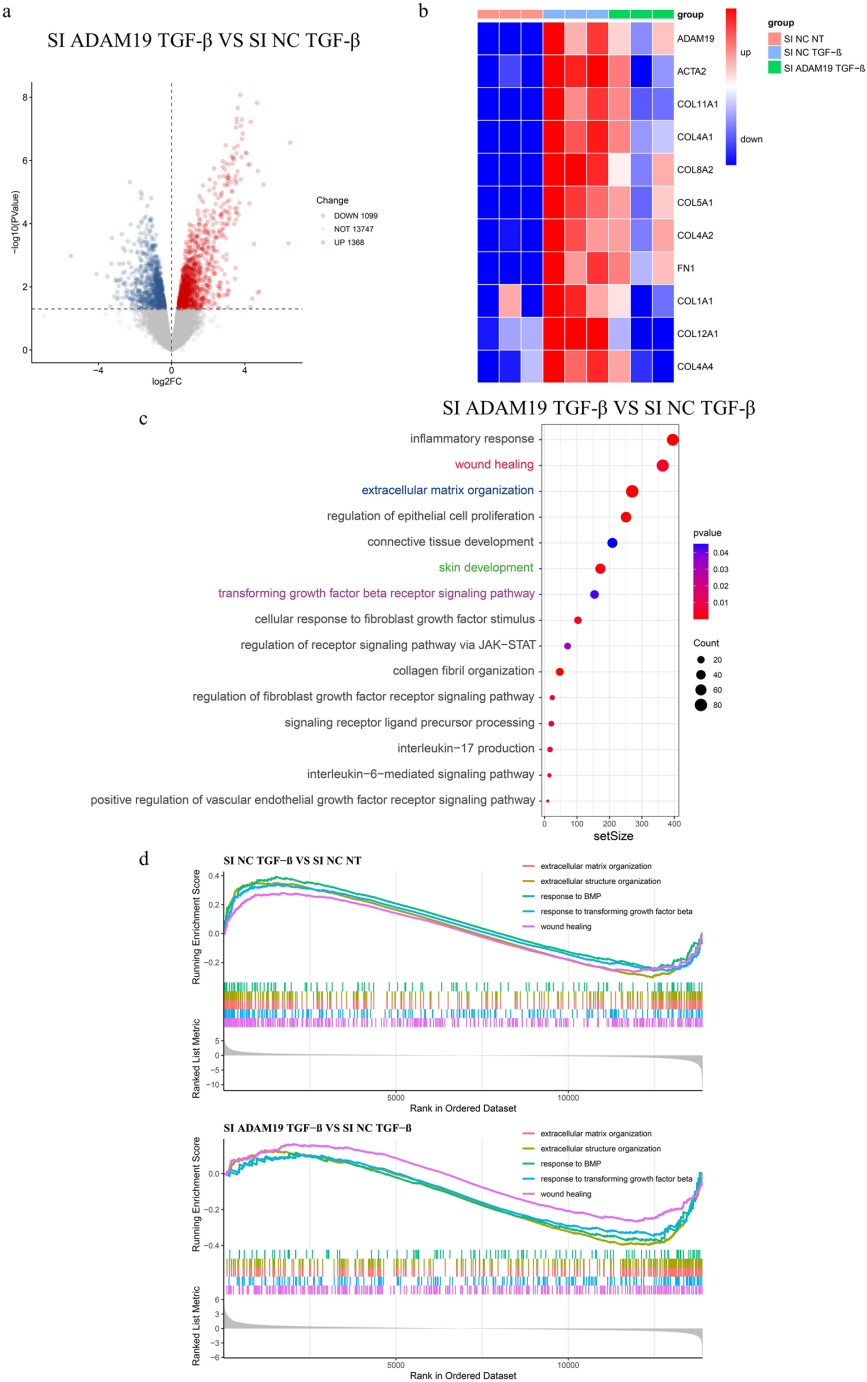
Knockdown of ADAM19 inhibited collagen formation and fibroblast activation. (**a**) Volcano plot of different expression genes (DEGs) between SI ADAM19 TGF-β and SI NC TGF-β. (**b**) Heatmap displaying ADAM19 and collagen genes expression in human primary dermal fibroblasts isolated from HC transfected with negative control siRNA and ADAM19 siRNA with or without TGF-β (10ng/ml) stimulation for 48 h. (**c**) GSEA between SI ADAM19 TGF-β and SI NC TGF-β. (**d**) Enrichment scores of GSEA related to fibroblast activation and fibrosis in Si NC TGF-β vs. Si NC NT and Si ADAM19 TGF-β vs. Si NC TGF-β. HC, healthy controls

To investigate the molecular mechanisms underlying the effects of ADAM19 on fibroblasts, we performed bulk RNA-seq analysis on cultured HC primary dermal fibroblasts that were transfected with negative control siRNA or ADAM19 siRNA, and further stimulated with or without TGF-β (Si NC NT, Si NC TGF-β, and Si ADAM19 TGF-β groups). We identified 2467 differentially expressed genes (1099 downregulated and 1368 upregulated with *P* < 0.05) in TGF-β-stimulated fibroblasts with and without ADAM19 knockdown (Fig. 4a). After TGF-β stimulation, many collagen genes, including COL1A1, COL11A1, COL4A1, COL8A2, COL5A1, COL4A2, COL12A1, and COL4A4, were upregulated, but their expression decreased upon inhibiting ADAM19 (Fig. 4b). Gene Set Enrichment Analysis (GSEA) between Si ADAM19 TGF-β and Si NC TGF-β revealed enrichments of various biological processes relevant to tissue fibrosis, such as “extracellular matrix organization,” “regulation of epithelial cell proliferation,” “collagen fibril organization,” “wound healing,” TGF-β related pathways, and others. We also observed the enrichment of several inflammatory-related pathways, including “inflammatory response,” “interleukin-6-mediated signaling pathway,” and “interleukin-17 production.” Interestingly, “signaling receptor ligand precursor processing” was enriched (Fig. 4c, Figure S4a). Furthermore, we compared the enrichment pathways between Si NC TGF-β vs. Si NC NT and Si ADAM19 TGF-β vs. Si NC TGF-β. Our analysis showed pathways in “wound healing,” “response to transforming growth factor beta,” “extracellular structure organization,” “response to BMP,” and “extracellular matrix organization” were increased in Si NC TGF-β vs. Si NC NT while decreased in Si ADAM19 TGF-β vs. Si NC TGF-β (Fig. 4d).

### The mechanisms of ADAM19 in TGF-β induced fibrosis

**Fig. 5:**
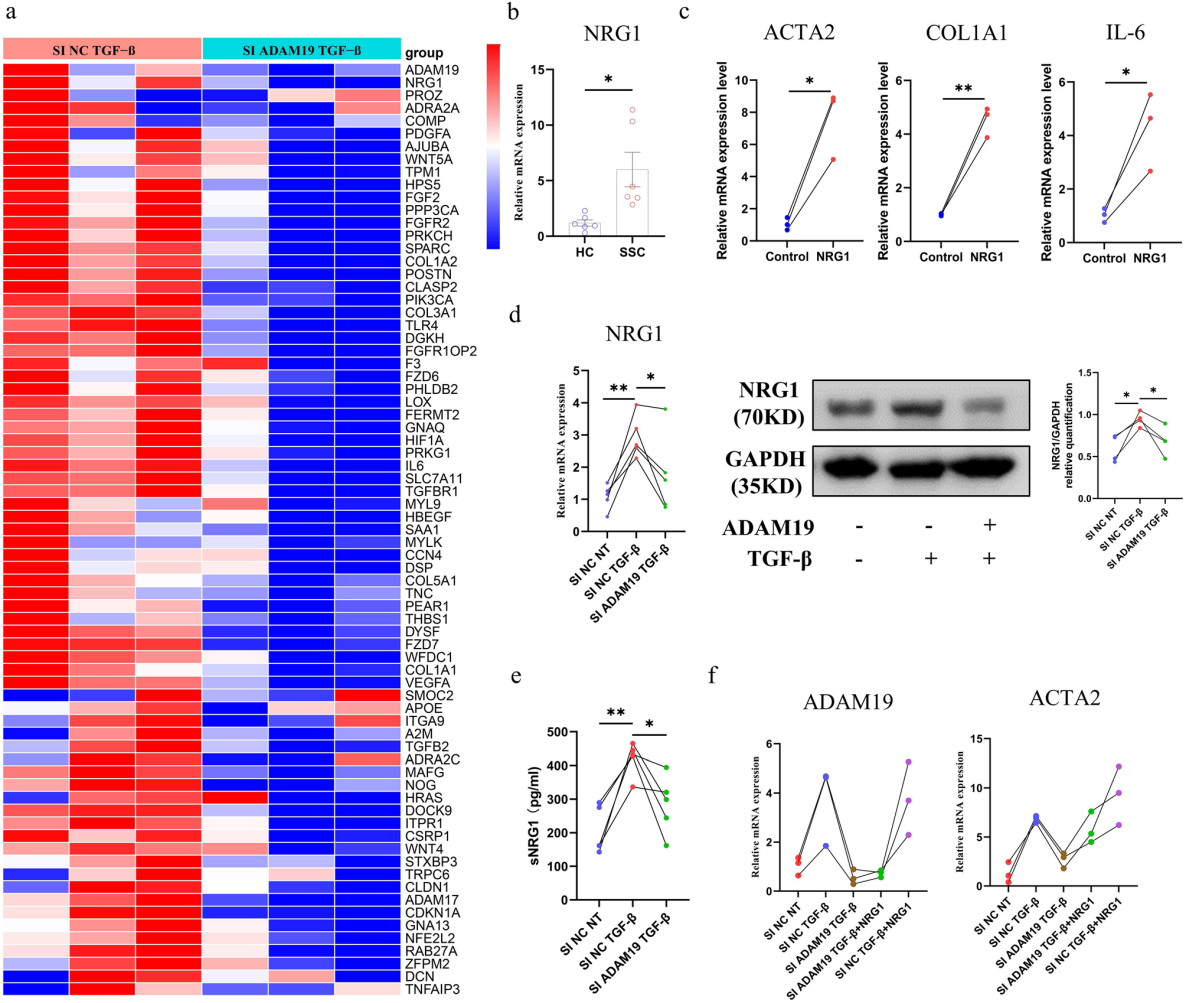
ADAM19 affects the expression of profibrotic cytokines NRG1 in human dermal fibroblasts. (**a**) Heatmap displaying ADAM19 and genes involved in wound healing pathway expression in human primary dermal fibroblasts isolated from HC transfected with ADAM19 siRNA with or without TGF-β (10ng/ml) stimulation for 48 h. (**b**) mRNA expression of NRG1 in isolated primary dermal fibroblasts from HC and SSc patients. (**c**) mRNA expression of COL1A1, ACTA2, and pro-inflammatory cytokine IL-6 in recombinant human NRG1 (50ng/ml) incubation with human primary dermal fibroblast isolated from HC 24 h. (**d**) mRNA and protein levels of NRG1 in human primary dermal fibroblasts isolated from HC transfected with negative control siRNA and ADAM19 siRNA with or without TGF-β (10ng/ml) stimulation for 48 h. (**e**) The expression of sNRG1 in the culture supernatant of human primary dermal fibroblasts isolated from HC transfected with negative control siRNA and ADAM19 siRNA with or without TGF-β (10ng/ml) stimulation for 48 h. (**f**) mRNA expression of ADAM19 and ACTA2 in human primary dermal fibroblasts isolated from HC transfected with negative control siRNA and ADAM19 siRNA, and then stimulated with or without TGF-β (10ng/ml) and recombinant human NRG1 (50ng/ml) stimulation. HC, healthy controls; SSc, systemic sclerosis. Data are represented as mean ± SEM. *, *P* < 0.05, **, *P* < 0.01

From the bulk RNA-seq data, we observed that the inhibition of ADAM19 expression significantly influences genes associated with the wound healing pathway, including NRG1, COL1A1, COL1A2, COL3A1, and IL6 (Fig. 5a). NRG1, a ligand for the ErbB family of receptor tyrosine kinases, plays a crucial role in the proliferation and migration of fibroblasts during wound healing. Additionally, we found that the mRNA expression of NRG1 was significantly increased in SSc primary dermal fibroblasts compared with HC (Fig. 5b). After incubation with recombinant human NRG1 for 24 h, HC primary dermal fibroblasts showed a significant upregulation of mRNA expression of COL1A1, ACTA2, and the pro-inflammatory cytokine IL-6 (Fig. 5c). NRG1 was noticeably induced by TGF-β, while its mRNA and protein expression decreased upon the knockdown of ADAM19. Soluble NRG1 (sNRG1) was significantly increased in the HC primary dermal fibroblasts culture supernatant following TGF-β stimulation. However, genetic knockdown of ADAM19 led to a reduction in TGF-β-induced sNRG1 production, indicating the role of ADAM19 in TGF-β-induced NRG1 shedding (Fig. 5d-e). Furthermore, incubation of HC primary dermal fibroblasts with recombinant human NRG1 reversed the impact of ADAM19 siRNA on the mRNA expression of ACTA2 (Fig. 5f).

## Discussion

ADAM19, a member of the ADAMs family, is expressed widely in somatic tissues (Edwards et al. 2008). This study found that ADAM19 was upregulated in skin tissues of SSc patients and HOCl-induced skin fibrosis mouse model, as compared to respective non-fibrotic control skin. Interestingly, ADAM19 had a significant positive correlation with fibrosis-related genes, local skin scores, mRSS, STPR, and the positive of ARA in patients with SSc. Furthermore, levels of ADAM19 were notably elevated in SSc primary dermal fibroblasts compared to HC, especially in the case of COL11A1^+^ fibroblasts. COL11A1^+^ fibroblasts can change the composition of the extracellular matrix and facilitate tumor invasiveness (Zhang et al. 2023). Recent research has suggested that COL11A1^+^ fibroblasts are associated with myofibroblasts, the principal collagen-producing cells and the driving force behind fibrosis (Tabib et al. 2021; Xue et al. 2022; Zhu et al. 2023).

The TGF-β signaling pathway plays a crucial role in skin fibrosis associated with SSc (Varga and Pasche 2009). Inhibition of ADAM19 expression reduces both epithelia-to-myofibroblast trans-differentiation (EMT) and inflammation in TGF-β-stimulated HK-2 cells (Qiu et al. 2022). Our findings indicate that TGF-β stimulation significantly increases ADAM19 expression in primary dermal fibroblasts isolated from HC. Moreover, knocking down ADAM19 reduces the profibrotic effects induced by TGF-β, as evidenced by decreased collagen synthesis and the transformation of fibroblast into myofibroblast. Additionally, RNA sequencing results revealed that HC primary dermal fibroblasts, which underwent ADAM19 knockdown after TGF-β stimulation, exhibited deregulation in various functional terms related to collagen fiber formation, skin development, ECM production, wound healing, and the TGF-β signaling pathway, in comparison to those without knockdown. These findings suggest that ADAM19 is involved in TGF-β-induced ECM deposition and fibroblast activation in skin fibrosis related to SSc.

Most ADAMs containing an active metalloprotease domain have the function of protein shedding, and many shed proteins are activated through cleavage, increasing the bioavailability of the protein (Huovila et al. 2005; Moss and Lambert 2002). The proteins shed by ADAMs include cytokines, growth factors, and growth factor receptors (Zunke and Rose-John 2017; Parkin and Harris 2009). ADAM17 was over-expressed in the skin of SSc patients and HOCl-induced mice with SSc. The role of ADAM17 in SSc skin fibrosis was related to its shedding of Notch receptors and activation of the Notch signaling pathway (Kavian et al. 2010). Most NRG1 isoforms are membrane-anchored ligands, cleaved by protease and secreted as soluble mature forms. This cleavage was essential for its signaling activity, which includes promoting fibroblast migration and releasing profibrotic cytokines (Horiuchi et al. 2005; Jumper et al. 2017; Kim et al. 2012). ADAM10, ADAM17, and ADAM19 are known to cleave NRG1 (Shirakabe et al. 2001; Luo et al. 2011). According to our results, NRG1 was significantly increased in SSc primary dermal fibroblasts. Recombinant human NRG1 induced in vitro activation of HC primary dermal fibroblasts. Inhibition of ADAM19 reduced TGF-β induced soluble NRG1 production in HC primary dermal fibroblasts. Moreover, recombinant human NRG1 incubation with HC primary dermal fibroblasts reversed the effect of ADAM19 siRNA on fibroblast activation. Therefore, we speculate that TGF-β promotes the expression of ADAM19, increasing the shedding of NRG1 in dermal fibroblasts and profibrotic activity.

Interstitial lung disease (ILD) is one of the most serve complications associated with SSc and can significantly affect mortality. The pathology is characterized by progressive lung fibrosis (Volkmann et al. 2022; Khanna et al. 2015). Although the lung is a prominent affected organ in SSc, its inaccessibility limits the widespread use of pulmonary tissue for clinical and research purposes. The mechanisms of SSc-associated ILD (SSc-ILD) remain elusive, and there is currently a lack of effective anti-fibrosis drugs for its treatment (Flaherty et al. 2019; King et al. 2014; Distler et al. 2019; Behr et al. 2021). Skin is another prominently affected organ, it’s easier and safer to obtain than pulmonary tissue. Shervin Assassi et al. first studied the skin transcripts and correlated them with the severity of ILD in the GENISOS cohort (Assassi et al. 2013). Here, we found that skin ADAM19 expression was significantly increased in SSc patients with ILD compared to those without ILD. Furthermore, SSc patients’ skin ADAM19 expression was negatively correlated with FVC and DLco, indicating that ADAM19 positively correlated with lung fibrosis severity. Studies showed that ADAM19 expression was upregulated in alveolar epithelial cells by TGF-β and played a role in the deposition of collagen and ECM in idiopathic pulmonary fibrosis, the most common type of ILD (Keating et al. 2006). Our study elucidated the functional role of ADAM19 in skin fibrosis associated with SSc. However, the mechanism of ADAM19 in SSc-ILD needs further study.

TGF-β is generally secreted from monocytes, lymphocytes, and fibroblasts in an inactive complex form that must be activated to exert functional effects. The latent TGF-β composed of bioactive TGF-β and latency-associated peptide (LAP) (Annes et al. 2003; Travis and Sheppard 2014). Serine proteases, thrombospondin, cell surface integrins, and matrix metalloproteinases have been reported to cleave LAP and active TGF-β (Umeda et al. 2021; Malenica et al. 2021; Bourd-Boittin et al. 2011). Studies found ADAM9-mediate shedding of LAP to produce bioactive TGF-β (Umeda et al. 2021). Similar to ADAM9, ADAM19 and LAP share the same integrin-binding ability and come into physical proximity (Qi et al. 2009; Mahimkar et al. 2005). Interestingly, functional analysis from the RNA sequencing data revealed that the knockdown of ADAM19 affects TGF-β associated pathways, including TGF-β production and cellular response to TGF-β. Further study is required to determine whether ADAM19 affects fibrosis by LAP cleaving and TGF-β activating, in other words, the positive feedback loop between ADAM19 and TGF-β.

In summary, ADAM19 plays a role in TGF-β-induced ECM deposition and fibroblast activation by mediating the shedding of NRG1, ultimately contributing to developing skin fibrosis in SSc. This study has several important limitations that should be addressed in future research (Prescott et al. 1992). At this stage, our investigation primarily centers around the pro-fibrotic roles of ADAM19 in isolated primary fibroblasts under in vitro conditions. To advance our understanding of ADAM19’s functions in a physiological context, it is crucial to explore its in vivo roles. This could be accomplished by utilizing conditional ADAM19 knockout mice, allowing us to assess whether ADAM19 could be a viable target for anti-fibrotic therapies (Fleischmajer et al. 1977). The interactions among immune cells, cytokines, and fibroblasts in the context of skin fibrosis associated with SSc are notably intricate. The roles of these components in the fibrotic process are still not fully understood. Therefore, further experimental investigations are necessary to clarify the role of ADAM19 and how it interacts with these different cell types in the skin of SSc patients. This understanding will be essential for identifying potential therapeutic strategies to mitigate fibrosis in these patients.

## Electronic supplementary material

Below is the link to the electronic supplementary material.

Supplementary Material 1

## Data Availability

The data used to support the findings of this study is available from GSE130955, GSE58095, GSE181549, GSE124161, and GSE138669. Primary data are available upon request.
