# Supplementary material for: ADAM Metallopeptidase domain 19 promotes skin fibrosis in systemic sclerosis via neuregulin-1
Source: Mol Med. 2024 Dec 23;30:269. doi: 10.1186/s10020-024-01047-8 (PMC11665244; doi:10.1186/s10020-024-01047-8)
Supplement: Supplementary file 1 — Supplementary Material 1 [file 10020_2024_1047_MOESM1_ESM.docx]

**Figure S1.** (a) ADAM19 gene expression levels in the skin of HC, SSc-non-ILD, and SSc-ILD in the GENISOS (GSE58095) cohort. HC, n = 43; Non-ILD, n = 36; ILD, n = 23. (b-c) Correlation between ADAM19 gene expression levels and DLco and FVC in the GENISOS (GSE58095) cohort. Correlation analysis was performed by Spearman’s method. HC, healthy controls; SSc, systemic sclerosis; ILD, interstitial lung disease. Data are represented as mean ± SEM. *, *P* < 0.05, ***, *P* < 0.001. ****, *P* < 0.0001.

**Figure S2.** (a) Volcano plots displaying ADAM19 and fibrosis-related genes (ACTA2, COL1A1, COL1A2, and FN1) expression in wound healing at weeks 1, 2, 3, 4, 5, 6, 8 (GSE124161). (b) Time course expression of ADAM19 and fibrosis-related genes (ACTA2, COL1A1, COL1A2, and FN1) in wound healing (GSE124161).


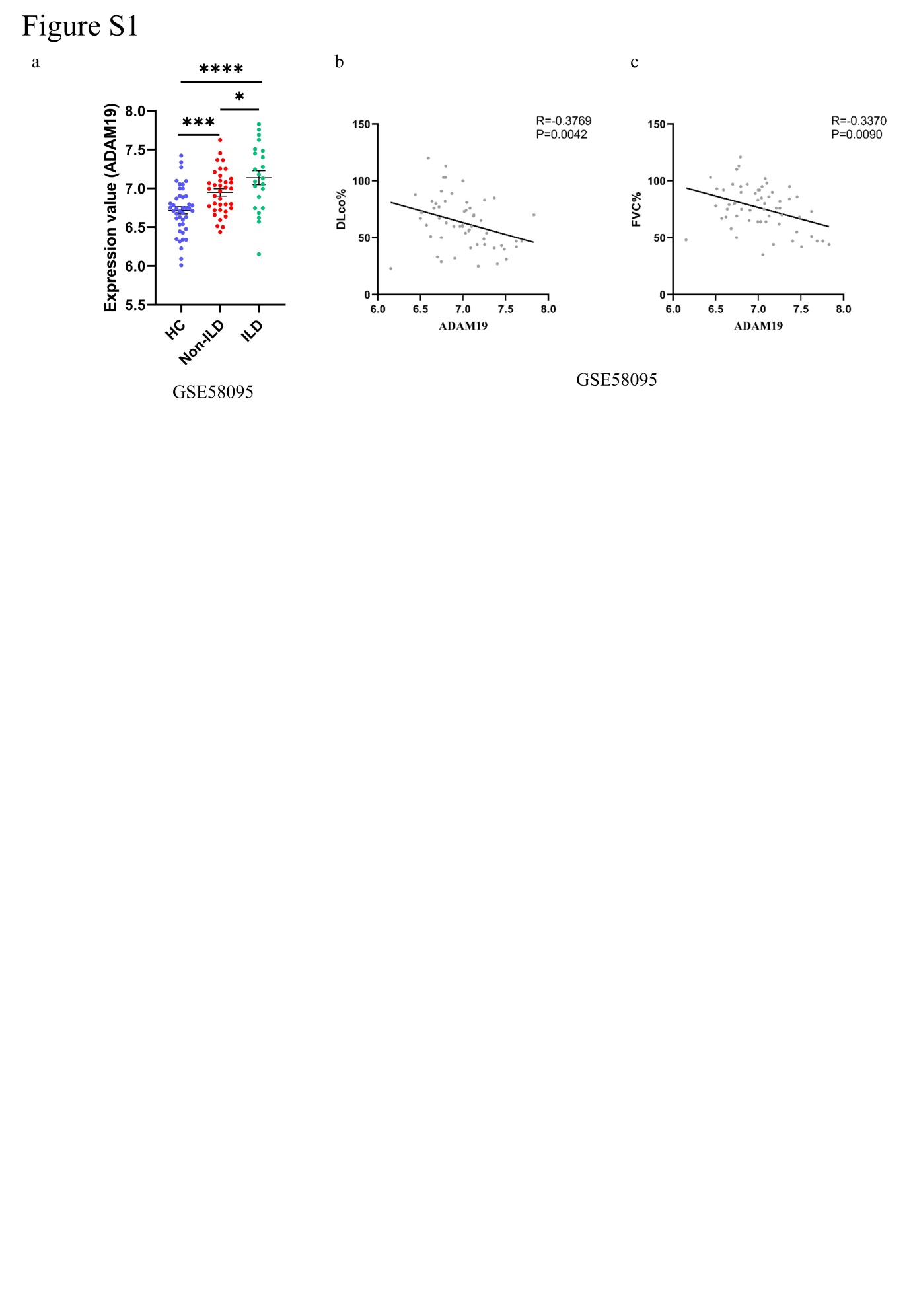

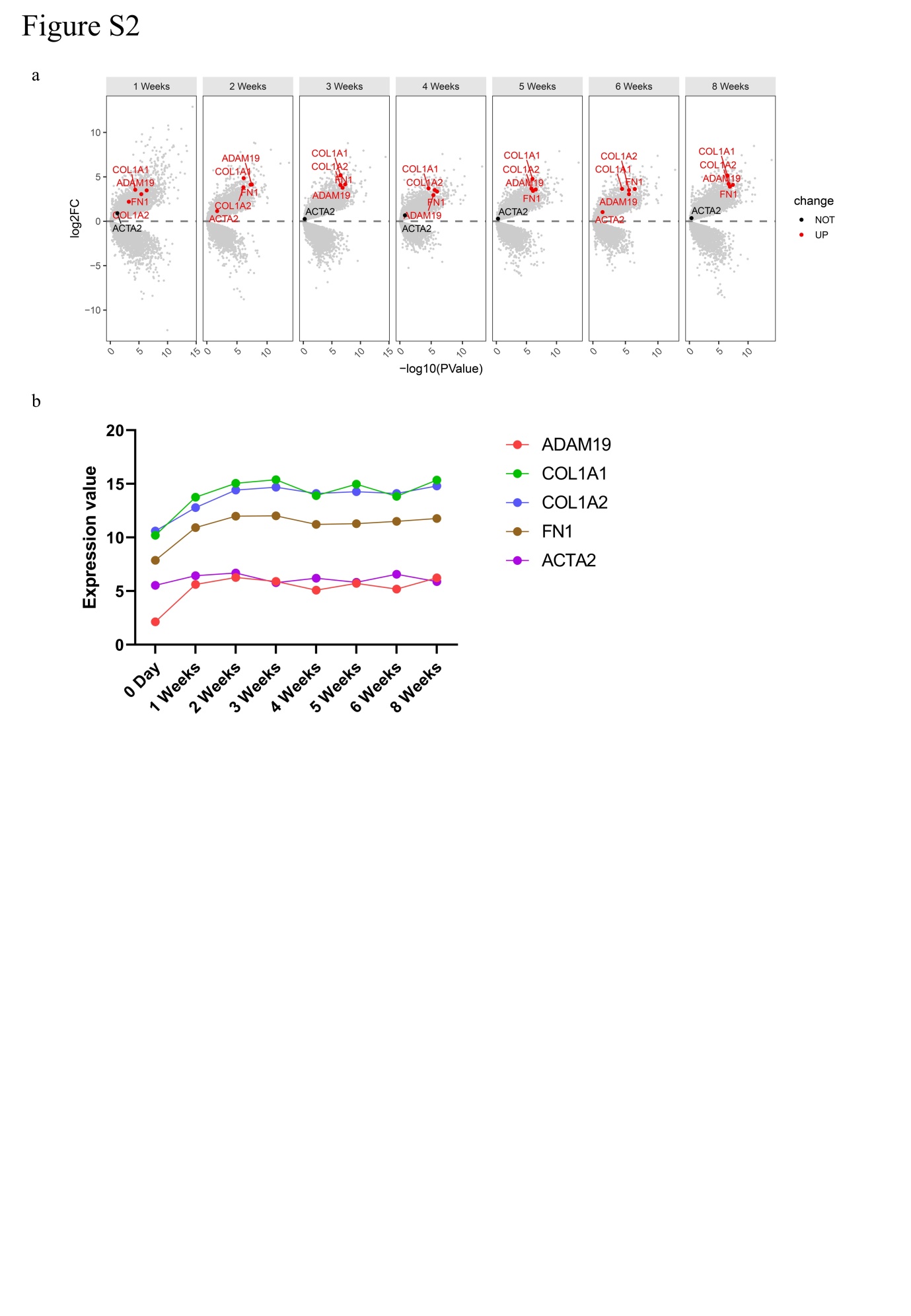


**Figure S3.** (a) ADAM19 gene expression in fibroblasts subpopulations of skin tissues of HC and SSc patients (GSE138669). HC, healthy controls; SSc, systemic sclerosis.


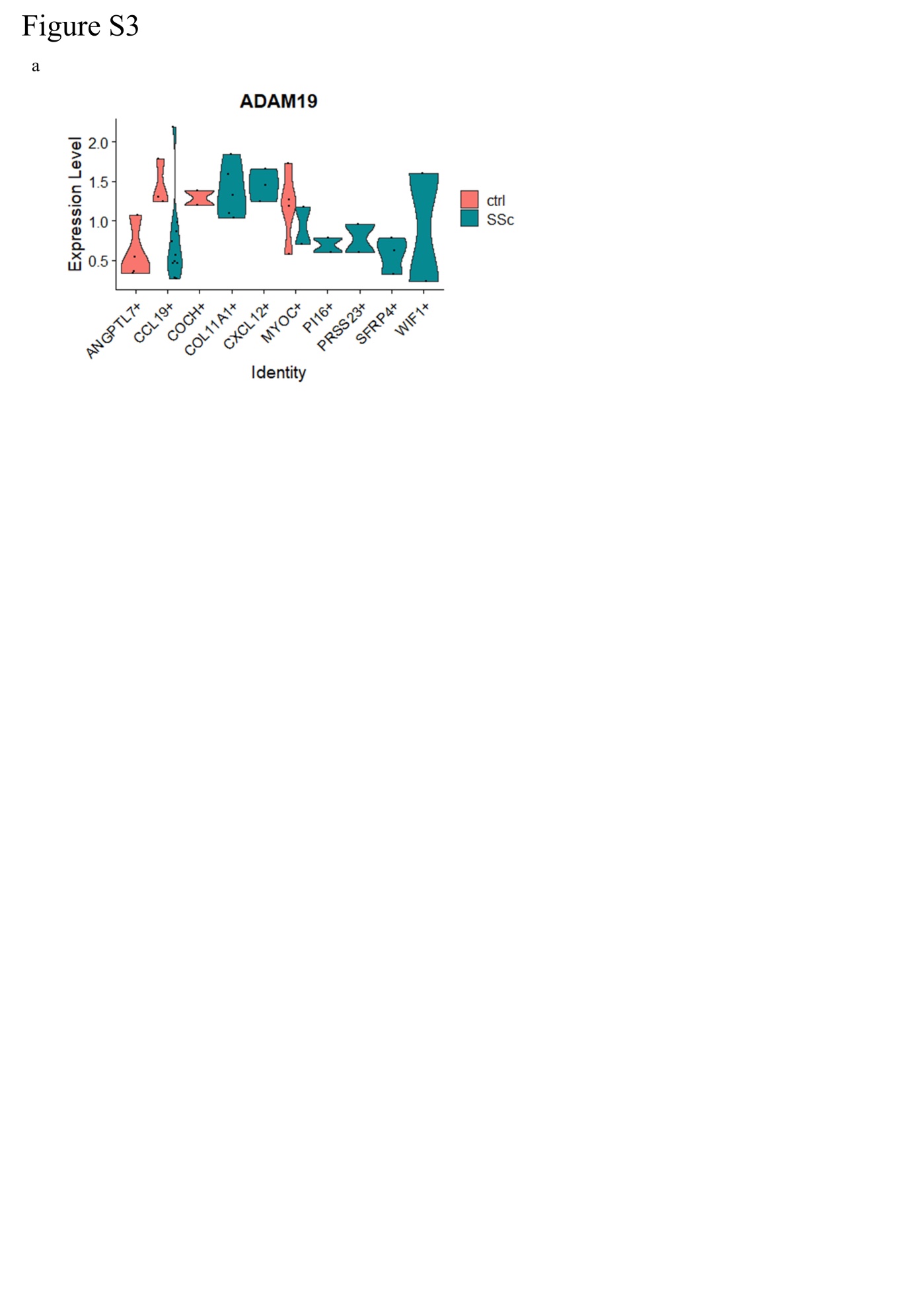


**Figure S4.** (a) Suppressed TGF-β related pathways in GSEA between TGF-β stimulated human primary dermal fibroblasts isolated from HC transfected with ADAM19 siRNA and negative control siRNA (Si ADAM19 TGF-β VS Si NC TGF-β). GSEA, Gene Set Enrichment Analysis; HC, healthy controls.


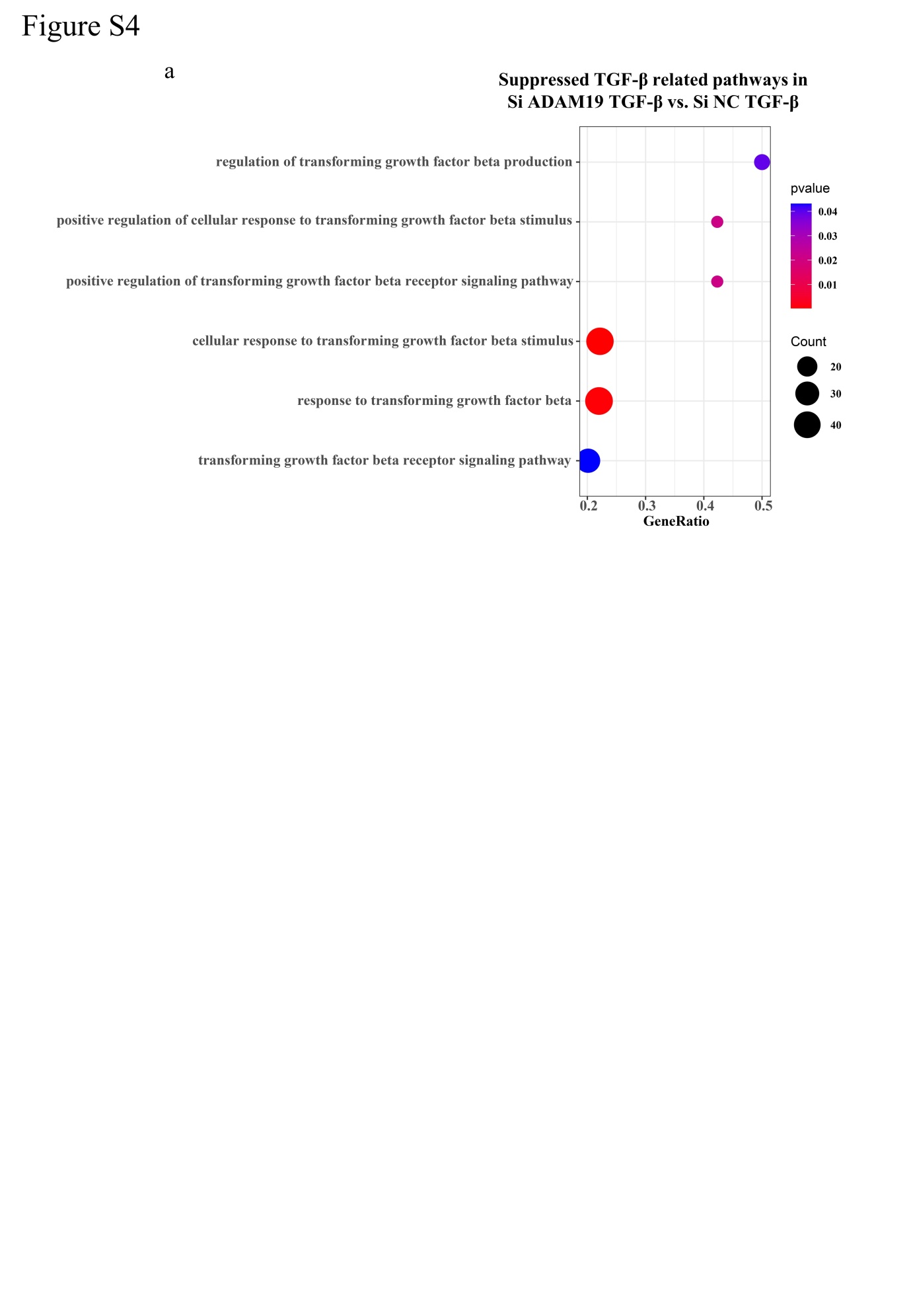
 **Supplementary Tables**

**Supplementary Table 1**: The clinical characteristics of SSc patients

| **Patient characteristics (n=6)** | |
| --- | --- |
| Female | 83.3 % |
| Age (median and range) | 42 years (20 – 64) |
| disease duration (median and range) | 18 months (3-36) |
| 2013 ACR/EULAR criteria fulfilled | 100 % |
| dcSSc | 50 % |
| SSc-ILD | 83.33 % |
| mRSS (median and range) | 12.5 (4-24) |
| PAH | 0 % |
| RP | 66.67 % |
| Ischemic ulcers at the time of biopsy | 16.67 % |
| History of ischemic ulcers | 50.0 % |
| Autoantibodies  ANAs  Anti-topoisomerase autoantibodies | 100 %  66.67 % |

**Supplementary Table 2**: Primer sequences

| **Primer** | **Species** | **Sequence** |
| --- | --- | --- |
| *ADAM19* forward | Human | 5’- CGAGAAGGTGAATGTGGCAGGA -3’ |
| *ADAM19* reverse | Human | 5’- AGCTCTGACACTGGATCTTCCC - 3’ |
| *ADAM19* forward | Mouse | 5’- GTGCCTCACTTACCAGGAACAG -3’ |
| *ADAM19* reverse | Mouse | 5’- GGACTGCACTTCCTGTATTGGC -3’ |
| *ACTA2* forward | Human | 5’- CTATGCCTCTGGACGCACAACT -3’ |
| *ACTA2* reverse | Human | 5’- CAGATCCAGACGCATGATGGCA -3’ |
| *ACTA2* forward | Mouse | 5’- TGCTGACAGAGGCACCACTGAA -3’ |
| *ACTA2* reverse | Mouse | 5’- CAGTTGTACGTCCAGAGGCATAG -3’ |
| *COL1A1* forward | Human | 5’- GATTCCCTGGACCTAAAGGTGC -3’ |
| *COL1A1* reverse | Human | 5’- AGCCTCTCCATCTTTGCCAGCA -3’ |
| *COL1A1* forward | Mouse | 5’- CCTCAGGGTATTGCTGGACAAC -3’ |
| *COL1A1* reverse | Mouse | 5’- CAGAAGGACCTTGTTTGCCAGG -3’ |
| *COL1A2* forward | Human | 5’- CCTGGTGCTAAAGGAGAAAGAGG -3’ |
| *COL1A2* reverse | Human | 5’- ATCACCACGACTTCCAGCAGGA -3’ |
| *COL1A2* forward | Mouse | 5’- TTCTGTGGGTCCTGCTGGGAAA -3’ |
| *COL1A2* reverse | Mouse | 5’- TTGTCACCTCGGATGCCTTGAG -3’ |
| *FN1* forward | Human | 5’- ACAACACCGAGGTGACTGAGAC -3’ |
| *FN1* reverse | Human | 5’- GGACACAACGATGCTTCCTGAG -3’ |
| *FN1* forward | Mouse | 5’- CCCTATCTCTGATACCGTTGTCC -3’ |
| *FN1* reverse | Mouse | 5’- TGCCGCAACTACTGTGATTCGG -3’ |
| *NRG1* forward | Human | 5’- GATTCCTACCGAGACTCTCCTC -3’ |
| *NRG1* reverse | Human | 5’ – TGGAAGGCATGGACACCGTCAT -3’ |
| *GAPDH* forward | Human | 5’- GTCTCCTCTGACTTCAACAGCG -3’ |
| *GAPDH* reverse | Human | 5’- ACCACCCTGTTGCTGTAGCCAA -3’ |
| *GAPDH* forward | Mouse | 5’- CATCACTGCCACCCAGAAGACTG -3’ |
| *GAPDH* reverse | Mouse | 5’- ATGCCAGTGAGCTTCCCGTTCAG -3’ |
